# Estimating Treatment Effect of Adjuvant Chemotherapy in Elderly Patients With Stage III Colon Cancer Using Bayesian Networks

**DOI:** 10.1200/CCI.23.00080

**Published:** 2023-09-25

**Authors:** Melle Sieswerda, Ruby van Rossum, Inigo Bermejo, Gijs Geleijnse, Katja Aben, Felice van Erning, Ignace de Hingh, Valery Lemmens, André Dekker, Xander Verbeek

**Affiliations:** ^1^ Department of Research and Development, Netherlands Comprehensive Cancer Organization, Utrecht, the Netherlands; ^2^ Department of Radiation Oncology (Maastro), GROW School for Oncology and Reproduction, Maastricht University Medical Centre+, Maastricht, the Netherlands; ^3^ Department for Health Evidence, Radboud University Medical Centre, Nijmegen, the Netherlands; ^4^ Department of Surgery, Catharina Hospital, Eindhoven, the Netherlands

## Abstract

**PURPOSE:**

While adjuvant therapy with capecitabine and oxaliplatin (CAPOX) has been proven to be effective in stage III colon cancer, capecitabine monotherapy (CapMono) might be equally effective in elderly patients. Unfortunately, the elderly are under-represented in clinical trials and patients included may not be representative of the routine care population. Observational data might alleviate this problem but is sensitive to biases such as confounding by indication. Here, we build causal models using Bayesian Networks (BNs), identify confounders, and estimate the effect of adjuvant chemotherapy using survival analyses.

**METHODS:**

Patients 70 years and older were selected from the Netherlands Cancer Registry (N = 982). We developed several BNs using constraint-based, score-based, and hybrid algorithms while precluding noncausal relations. In addition, we created models using a limited set of recurrence and survival nodes. Potential confounders were identified through the resulting graphs. Several Cox models were fitted correcting for confounders and for propensity scores.

**RESULTS:**

When comparing adjuvant treatment with surgery only, pathological lymph node classification, physical status, and age were identified as potential confounders. Adjuvant treatment was significantly associated with survival in all Cox models, with hazard ratios between 0.39 and 0.45; CIs overlapped. BNs investigating CAPOX versus CapMono did not find any association between the treatment choice and survival and thus no confounders. Analyses using Cox models did not identify significant association either.

**CONCLUSION:**

We were able to successfully leverage BN structure learning algorithms in conjunction with clinical knowledge to create causal models. While confounders differed depending on the algorithm and included nodes, results were not contradictory. We found a strong effect of adjuvant therapy on survival in our cohort. Additional oxaliplatin did not have a marked effect and should be avoided in elderly patients.

## Teaser

Advanced statistical analysis (with BNs) suggests that oxaliplatin should be avoided in elderly patients.

## INTRODUCTION

With over 8,000 new cases annually, which constitutes approximately 7% of the total cancer incidence, colon cancer is the fourth most common type of cancer in the Netherlands.^1^ Given that treatment is always a balance between cost (both financial and physical) and benefit, it is important to continuously assess and improve the effect of (adjuvant) treatment.

**CONTEXT**

**Key Objective**
Adjuvant chemotherapy in elderly is effective although previous analyses dispute the benefit of adding oxaliplatin to fluoropyrimidines. Using observational data to estimate treatment effect always comes with the risk of bias, specifically confounding by indication. Here, we use structure learning algorithms for Bayesian Networks, in conjunction with clinical knowledge, to identify confounders, mitigate this risk, and reliably estimate the effect of adjuvant treatment in colon cancer.
**Knowledge Generated**
Structure learning can aid in finding causal relationships in observational data by precluding noncausal relationships, thus facilitating identification of confounders. We found a strong effect of adjuvant therapy on survival in elderly patients (70 years and older); additional oxaliplatin provided no further benefit.
**Relevance**
Identification of and correction for confounders is required for using observational data for estimating treatment effects. The ability to do so greatly enhances the use of observational data. Our research suggests oxaliplatin should be avoided in elderly patients.

For stage III colon cancer, Dutch guidelines recommend treatment with surgery and adjuvant chemotherapy with either capecitabine and oxaliplatin (CAPOX) or infusional folinic acid-fluorouracil-oxaliplatin (FOLFOX). However, in elderly patients, the benefit of oxaliplatin is still a point of contention.^2,3^

While 59% of the patients with colon cancer are older than 70 years, elderly patients are frequently excluded from clinical trials on the basis of age alone. Even if age does not rule out participation, elderly patients included may still not be representative for daily clinical practice as they must be fit enough to satisfy other inclusion criteria.

To bridge this gap, observational data play a vital role. Unfortunately, when it comes to estimating treatment effect, observational data are sensitive to bias, specifically selection biases like confounding by indication. For example, if predominantly relatively healthy patients receive treatment, this will skew results in favor of treatment.

There are different ways to account for confounding by indication, for example, through including confounders as covariates (in regression analysis), stratification, or propensity score correction.^4-9^ In any case, correction requires a decision on which variables should be included as confounders. Several criteria exist to help in this decision, for example, the pretreatment criterion, common cause criterion, backdoor path criterion, and disjunctive cause criterion.^10-12^ The pretreatment criterion would select *all* pretreatment variables as confounders. The common cause criterion would, as the name suggests, only correct for those variables thought to be common causes of exposure and outcome. The backdoor path criterion resembles the common cause criterion but takes chains of influence into account and can thus correct for indirect confounders. The disjunctive cause criterion would correct for those variables thought to be either a cause of exposure or outcome.

**FIG 1.**
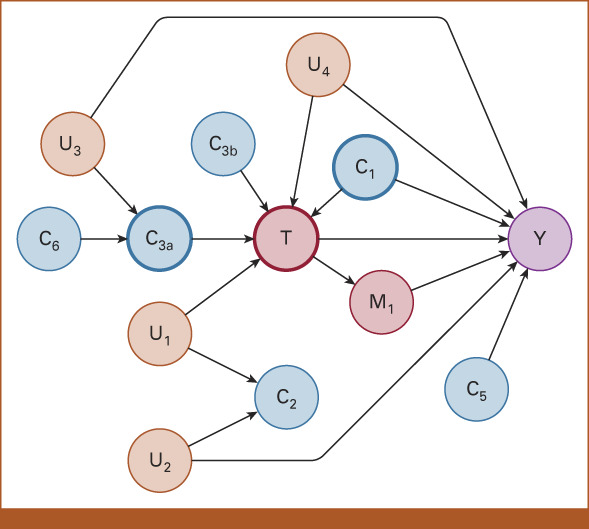
Different types of pathways between treatment (T) and outcome (Y). Blue nodes (C_⁎_) represent measured variables that could be considered potential confounders, and orange nodes (U_⁎_) represent unmeasured variables. Correcting for nodes C_1_ and C_3a_ is required to obtain an unbiased result. Correction for C_3a_ and C_6_ simultaneously would open the backdoor path through U_3_ and thus should be avoided. Correction for unmeasured variables U_1_ and U_2_ is unnecessary. Adjusting for C_2_ would open a backdoor path between T and Y, thus biasing the estimate. Correcting for C_3b_ and/or C_5_ would be an unnecessary, bias-neutral adjustment. Correction for M_1_ would yield unpredictable results, ranging from overestimating, to nullifying, to reversing any estimated effect. U_4_ is the only unmeasured confounder that cannot be corrected for. Figure adapted from VanderWeele and Shpitser^12^ (Fig 1).

When there is uncertainty about the presence of unmeasured confounding, it can be reasoned that, generally, the disjunctive cause or backdoor path criteria yield the most unbiased results. For example, correcting for variable C_2_ in Figure 1, which would be corrected for when applying the pretreatment criterion, would unnecessarily bias the estimated effect of T→ Y. Application of these criteria, however, requires a causal model. Interestingly, structure learning algorithms for Bayesian Networks (BNs) can be used—with input from domain experts—to discover causal models from data, as recently shown.^13^

Briefly, BNs are a type of probabilistic graphical model whose structure is determined by a directed acyclic graph where nodes represent variables and directed edges signify (preferably causal) relationships.^11,14^ Each node is associated with a probability distribution conditional on its parents (ie, nodes with a directed edge toward that node).

Structure learning algorithms for BNs can be classified into three categories. Constraint-based algorithms (eg, Necessary Path Condition [NPC]^15^) leverage the presence of conditional (in)dependencies to determine a model that best fits the data, score-based algorithms (eg, Silander-Myllymaki [SM]^16^) optimize a heuristic that describes goodness of fit, and hybrid algorithms (eg, Max-Min Hill-climbing [MMHC]^17^) combine constraint- and score-based strategies.

Generally, these algorithms can incorporate previous (clinical) knowledge in the form of mandatory and prohibited (directed) edges. While causal relations (mandatory edges) between variables may be contested, counterfactuals (prohibited edges) can often be supplied with reasonable certainty and consensus. For example, tumor characteristics, treatment, or outcome will never *cause* age even if the reverse relation (eg, age → tumor characteristics) is contended.

When using structure learning in conjunction with these expert-defined counterfactuals (a blacklist consisting of prohibited edge directions), it is possible to obtain a causal graph. This model can then be used for confounder identification by applying one of the aforementioned criteria and subsequently mitigation.

Previously, we analyzed the effect of adjuvant chemotherapy with CAPOX or capecitabine monotherapy (CapMono) in elderly patients with stage III colon cancer using data from the Netherlands Cancer Registry (NCR) which covers the entire Dutch population.^2^ In this analysis, we adjusted for confounding by indication using the pretreatment criterion.

In this article, we again investigate the treatment effect of adjuvant chemotherapy and the added benefit of oxaliplatin (CAPOX) over CapMono. To this end, we first build causal models using BNs in conjunction with different structure learning algorithms and a varying number of recurrence/survival nodes. Finally, we explore the effect of correcting for different sets of confounders, as identified by these causal models, and compare this with propensity score correction.

## METHODS

### Data

The original data set from the study by van Erning et al^2^ was used, and vital status was updated until January 2021. The median follow-up for overall survival (OS) was 58 months (after surgery) for all patients and 113 months for patients alive at last follow-up. The median follow-up for recurrence-free survival was 19 months (after surgery) and 29 months for patients alive at last follow-up.

Briefly, a cohort of patients, diagnosed in the southeastern part of the Netherlands between 2005 and 2012 with pathologic stage III (pT_1-4_N_1-2_M_0_) colon cancer and 70 years and older, was selected from the NCR. The variables sex, age, ASA classification (a system indicating physical performance status, developed by the American Society of Anesthesiologists), pT, pathological lymph node classification (pN), tumor subsite (coded according to ICD-O-3), and differentiation grade were included. In addition, in 2013 and 2014, details regarding adjuvant therapy, the number of comorbidities, and development of (local) recurrence were acquired from medical records and added to the NCR. The year of diagnosis was available but dropped after a quick analysis of its association with recurrence (Data Supplement, Variable Selection and Fig S1).

**FIG 2.**
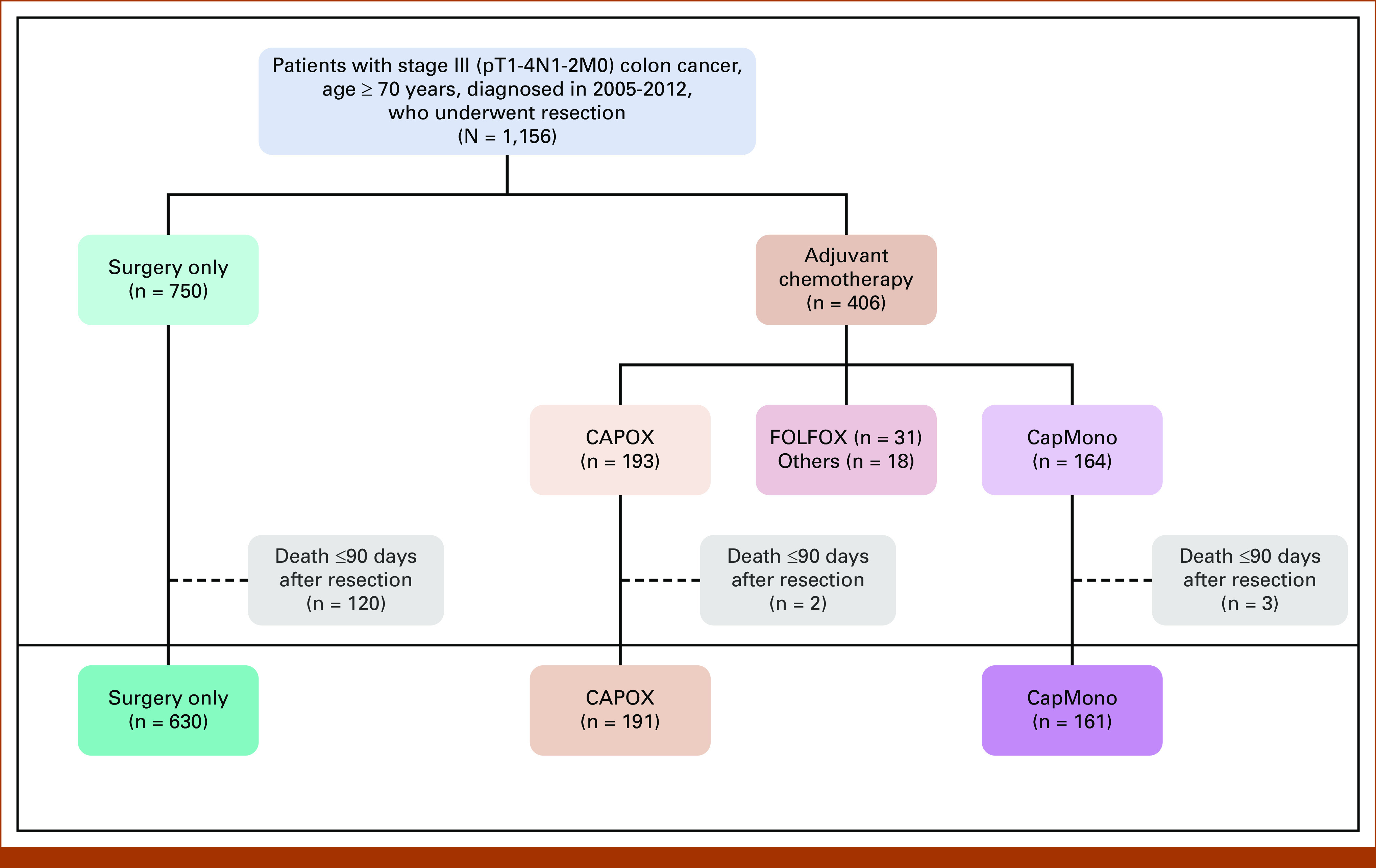
CONSORT diagram—overview of patients included in the study. CapMono, capecitabine monotherapy; CAPOX, capecitabine and oxaliplatin; FOLFOX, infusional fluorouracil, leucovorin, and oxaliplatin.

As before, patients who died within 90 days after surgery (n = 125) were excluded since these deaths were likely due to surgical complications and patients were unable to undergo adjuvant chemotherapy. Patients receiving chemotherapy other than CAPOX or CapMono (eg, FOLFOX) were excluded. This left a final data set consisting of n = 982 records (Fig 2; Table 1, Data Supplement, Tables S1 and S2). For analysis of CAPOX compared with CapMono, a subset consisting of patients who received adjuvant therapy was created (n = 352).

**TABLE 1.**
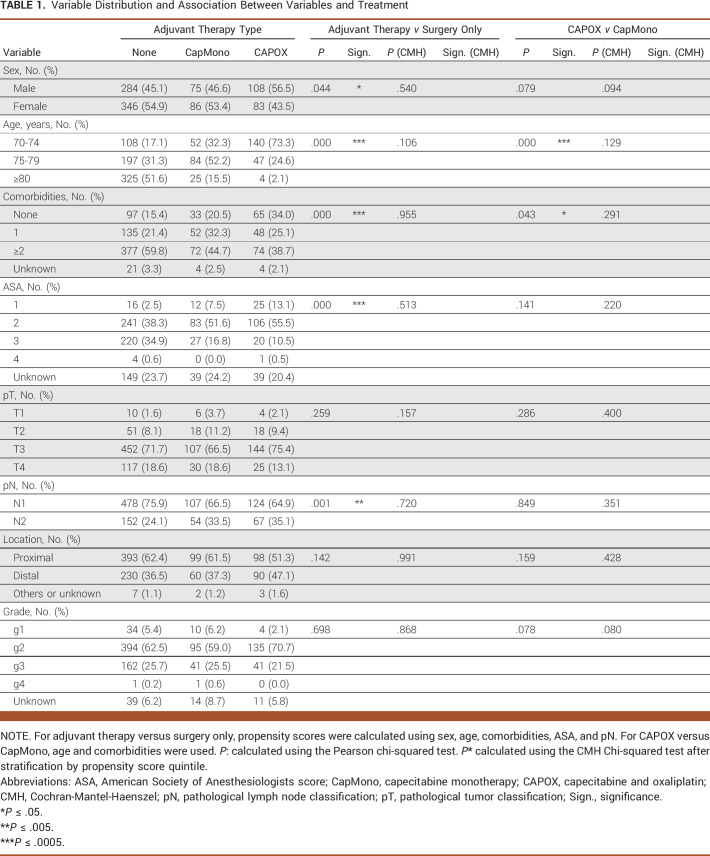
Variable Distribution and Association Between Variables and Treatment

For assessing association with treatment and for use in the BN, age was discretized into three categories: 70-74, 75-79, and 80 years and older (Data Supplement, Discretization of Age and Fig S2). The anatomic subsite was relabeled as proximal (caecum—splenic flexure of colon), distal (descending—sigmoid colon), or unknown/unspecified.

OS, counted from the date of surgery, was discretized into five Boolean variables representing 1- to 5-year survival, introducing not applicables (NAs; missing values) for patients who had a follow-up of <5 years and were alive at the time of follow-up. The absence of recurrence was discretized similarly. However, because of the limited follow-up, variables representing the absence of recurrence for ≥3 years were skewed toward recurrence or NA. Consequently, these three variables were dropped (Data Supplement, Variable Selection).

### Statistical Analyses

#### BN Development

Using different combinations of outcome variables, full and simplified BNs were developed. The full networks used two nodes/variables to represent recurrence at 1 and 2 years and five nodes for OS at 1, 2, 3, 4, and 5 years. The simplified networks used the last node of each.

The rationale for also evaluating a simplified network is the following. Having multiple outcome nodes at sequential time points might limit the BN ability to find other associations. For example, death at 1 year after diagnosis perfectly predicts death for all subsequent nodes/time points. Therefore, when testing for association between any other variable and a survival node at a later time point, one is essentially testing for association with survival in a specific interval. It can be reasoned that this reduces the (statistical) power to find associations with later time points. For example, if variable 1 is strongly associated with 1-year survival than any, association of variable 2 with 2-year survival will be harder to detect. In the simplified networks, only the final time point is included to obtain the maximal number of associations with the highest statistical power as the treatment effect on survival is expected to be the highest at that time point.

In addition, we evaluated the effect of using different algorithms than the constraint-based NPC algorithm, as available in Hugin.^18^ Specifically, we applied MMHC (hybrid) and SM (score-based) as implemented in the R package bnstruct.^16,17,19,20^ Either adjuvant treatment (Yes, No) or adjuvant treatment regimen (CAPOX, CapMono) was included as appropriate. Obtained BNs were visualized using the R package qgraph.^21^

**FIG 3.**
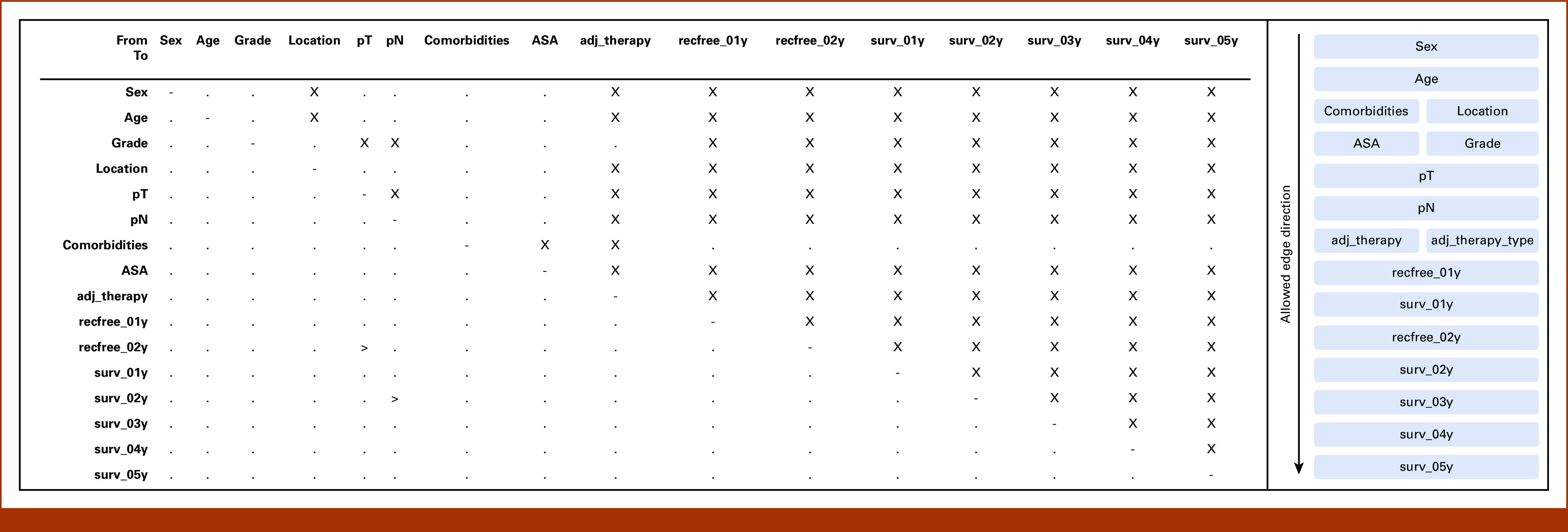
Structure learning constraints for the effect of adjuvant chemotherapy on overall survival. Left: constraints for Hugin. The blacklist is made up from cells containing x: these edges are forbidden. Minimal whitelist (ie, enforced edges) is formed by cells marked with >. Right: constraints for bnstruct. Edges can only originate from a node in a layer at the same height or higher. For example, sex → age is allowed, but not the reverse. Edges between nodes at the same height can point either way. ASA, American Society of Anesthesiologists score; pN, pathological lymph node classification; pT, pathological tumor classification.

In all cases, structure learning was performed with the level of significance set to 0.05.^15^ In Hugin, the process was supported using a manually defined blacklist of prohibited edge directions (Fig 3). bnstruct uses a different, less granular approach and accepts a list of layers, where edges from lower to higher layers are forbidden.

The NPC algorithm may yield ambiguous regions, consisting of a set of interdependent uncertain links where the absence of one link depends on the presence of another. When this happens, Hugin prompts for input. For resolution, we first prioritized associations with outcome nodes (recurrence and survival) and subsequently associations with treatment.

In some situations, Hugin ignores the supplied blacklist after manual resolution and misdirects the selected edge. For example, Hugin created an edge from the node representing 4-year survival (surv_04y) toward the ASA node. To overcome this issue, misdirected edges were added to a (minimal) whitelist of enforced edges and structure learning was repeated.

The resulting graphs were inspected to identify confounders.

#### Propensity Score Analysis

Propensity scores were calculated as follows. First, the chi-squared test was used to assess the association between pretreatment variables and adjuvant treatment. Next, variables with a significant association with treatment were used as covariates in the propensity score calculation. For adjuvant treatment versus surgery only, these were sex, age, comorbidities, ASA, and pN. For CAPOX versus CapMono, these were age and comorbidities only.

Subsequently, the propensity score was discretized using quintiles. Finally, the Cochran-Mantel-Haenszel (CMH) chi-squared test, with the bins as strata, was used to evaluate the effect of propensity score correction.^8,9,22^

#### Cox Proportional Hazards Models

**FIG 4.**
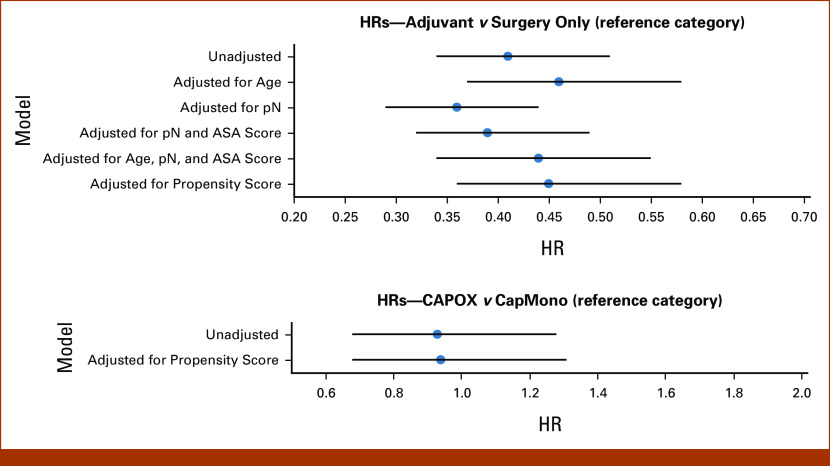
HRs (with CIs). Top: adjuvant treatment versus surgery only (referent category). Bottom: CAPOX versus CapMono (referent category). ASA, American Society of Anesthesiologists score; CapMono, capecitabine monotherapy; CAPOX, capecitabine and oxaliplatin; HR, hazard ratio; pN, pathological lymph node classification.

Several Cox models were developed, with each investigating the effect of (regimen of) adjuvant treatment on survival while correcting for a different set of potential confounders, driven by the results from structure learning. Covariates included are listed in Figure 4. Hazard ratios (HRs) for treatment were extracted, together with their CIs.

## RESULTS

### Adjuvant Treatment Versus Surgery Only

#### Variable Distribution and Propensity Score Correction

Chi-squared analysis showed that variables age, sex, pN, ASA (physical performance status), and comorbidities were significantly associated with receiving any form of adjuvant treatment (Table 1; Data Supplement, Table S1). Stratification using the discretized propensity score as strata in the CMH chi-squared test removed all associations between these conceivable confounders and treatment.

#### BN Development and Confounder Identification

**FIG 5.**
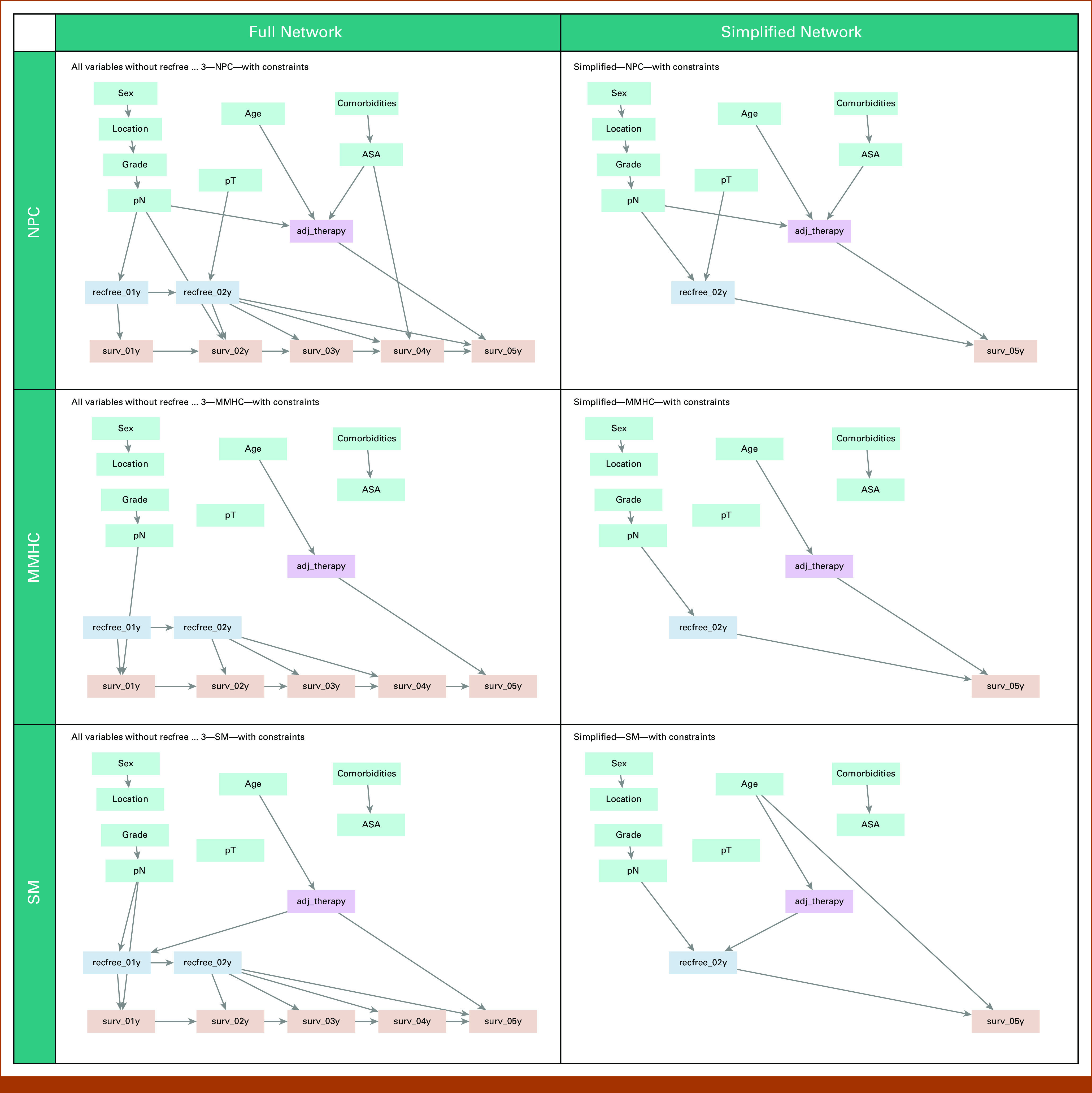
Bayesian Networks for adjuvant treatment versus surgery only. Rows 1-3 correspond to the algorithms NPC, MMHC, and SM, respectively. The left column shows full networks, and the right column shows simplified networks. Treatment indicates whether any form of adjuvant treatment was received. Top left: pN and ASA act as confounders. Top right: pN acts as a confounder. Middle left: no confounders identified; pN, ASA, and treatment are independent. Middle right: no confounders identified. Bottom left: no confounders identified; pN, ASA, and treatment are independent. Bottom right: age acts as a confounder. ASA, American Society of Anesthesiologists score; MMHC, Max-min Hill-climbing; NPC, Necessary Path Condition; pN, pathological lymph node classification; pT, pathological tumor classification; SM, Silander-Myllymaki.

The full BNs are shown in the left column of Figure 5. In the BN found by the NPC algorithm, two confounders were identified through inspection of the graph: pN and ASA. The BNs found by MMHC and SM algorithms did not identify any confounders and generally had fewer edges.

In the simplified networks, which focused on a limited set of recurrence and survival nodes (Fig 5, right column), the NPC algorithm again identified pN as confounder, but ASA, which was associated with surv_04y in the full network, was no longer associated with survival. The SM algorithm did not find an association between pN and treatment (node adj_therapy) but *did* find an association between age and surv_05y, identifying age as a potential confounder.

In summary, depending on the algorithm selected and nodes included, treatment was either unconfounded or confounded by pN, pN and ASA, or age.

#### Cox Proportional Hazards Models

Adjuvant treatment was significantly associated with survival in all models (Fig 4, top row; Data Supplement, Table S3). Estimated HRs were between 0.39 and 0.45, depending on covariates included. The models had overlapping CIs and yielded comparable results.

### CAPOX Versus CapMono

#### Variable Distribution and Propensity Score Correction

Chi-squared analysis showed variables age and comorbidities to be significantly associated with the choice between CAPOX or CapMono (Table 1; Data Supplement, Table S2). Here too, stratification using the binned propensity score as strata in the CMH chi-squared test removed all associations between these suspected confounders and treatment.

#### BN Development and Confounder Identification

**FIG 6.**
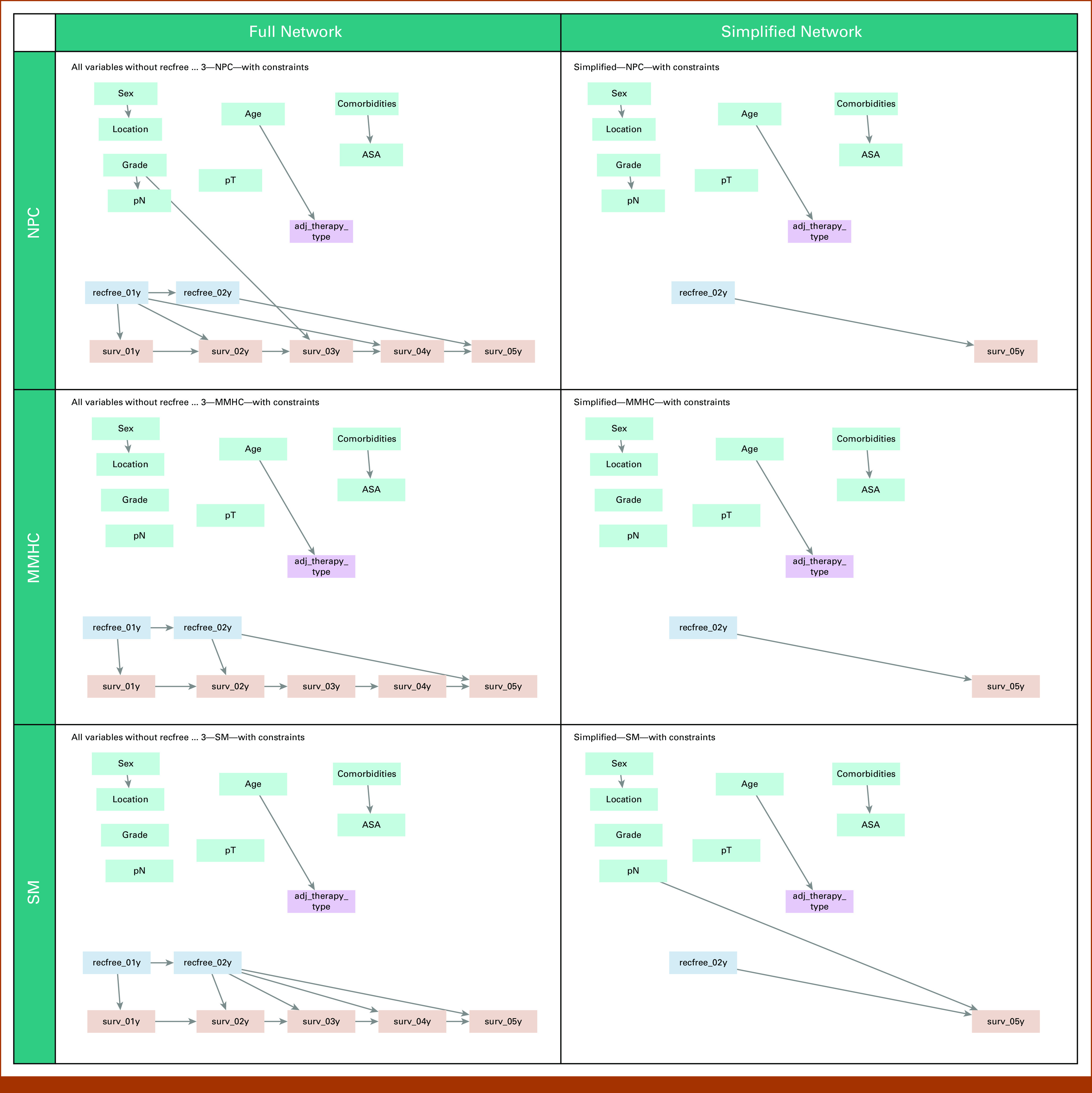
Bayesian Networks for CAPOX versus CapMono. Rows 1-3 correspond to the algorithms NPC, MMHC, and SM, respectively. The left column shows full networks, and the right column shows simplified networks. Treatment indicates whether CAPOX or CapMono was received. Top left: no confounders found. Top right: no confounders found. Middle left: no confounders found. Middle right: no confounders found. Bottom left: no confounders found. Bottom right: no confounders found. ASA, American Society of Anesthesiologists score; CapMono, capecitabine monotherapy; CAPOX, capecitabine and oxaliplatin; MMHC, Max-min Hill-climbing; NPC, Necessary Path Condition; pN, pathological lymph node classification; pT, pathological tumor classification; SM, Silander-Myllymaki.

From the full networks, none of the algorithms identified an association between the adjuvant treatment regimen and survival (Fig 6). From the pretreatment variables, only grade was associated with survival (using the NPC algorithm) and no confounders were found.

In the simplified networks, again, none of the algorithms identified an association between the adjuvant treatment regimen and survival. All networks suggested that age was a driving factor for the choice of regimen. The SM algorithm implied that pN was an independent predictor of survival.

To summarize, regardless of the algorithm selected and nodes included, choice of treatment regimen was not only unconfounded but also unassociated with survival.

#### Cox Proportional Hazards Models

None of the Cox models found significant association between the adjuvant treatment regimen and survival with HR point estimates around 0.93 and CIs equal to or wider than 0.68-1.28 (Fig 4, bottom row; Data Supplement, Table S4). CIs between the models were largely overlapping.

## DISCUSSION

We were able to successfully leverage BN structure learning algorithms in conjunction with (basic) clinical knowledge to create causal models and subsequently identify potential confounders. Different structure learning algorithms identified different potential confounders, but none were contradicting. In other words, confounders identified in one model were not mediators or colliders in others (Data Supplement, Fig S3), which may introduce bias if corrected for. From a theoretical point of view, score-based algorithms run a higher risk of incorrectly classifying a variable as a confounder in the presence of *unmeasured* confounding (Data Supplement, Fig S4). In this case, this mechanism does not seem to play a significant role since all HRs were very close together.

When comparing adjuvant treatment with surgery only, the set of identified confounders differed, depending on both the specific algorithm used and the nodes included in the network. Potential confounders identified were pN, ASA, and age although 3 (out of 6) networks suggested that the treatment effect was unconfounded. All Cox models found significant benefit of adjuvant treatment over surgery only (HRs ranging between 0.36 and 0.46); correcting for different sets of potential confounders had no marked effect, and neither did correction using propensity score.

In the comparison between CAPOX and CapMono, no confounders were identified, regardless of the algorithm or nodes included: none of the BNs found an association between the choice of treatment regimen and survival. The unadjusted Cox model yielded the same conclusion and did not find any effect of adding oxaliplatin. Correcting for propensity score did not make a difference.

Looking at the BNs in general, several interesting associations between pretreatment variables are found in all networks, regardless of algorithm or node selection. Unsurprisingly, age appears to be associated with the choice of adjuvant treatment, not only when considering whether to start either regimen but also when making a choice for a specific regimen. Similarly, sex was associated with the tumor location and grade with pN, both associations that have been reported before.^23,24^ The association found between the number of comorbidities and ASA score also makes sense considering the use of the ASA score (physical performance status) to approximate comorbidity in registries.^25^ On the other hand, some expected associations were not, or only infrequently, found: only one network connected pT and survival. However, this might be explained by the fact that we selected stage III and the (vast) majority of patients had a pT3 tumor. Overall, we considered the obtained networks clinically plausible.

Previously, Wilkinson et al^26^ investigated the effect of adjuvant 5-fluorouracil with leucovorin compared with surgery only. The HR found here is smaller than the effect they reported, which was 0.62 in stage II-III and 0.65 in stage III only. It should be noted that the age distribution between our study (100% for patients older than 70 years) and the study by Wilkinson et al (approximately 36%-51% patients younger than 60 years depending on the treatment group) is vastly different. Our results seem to suggest that, in our elderly cohort, chemotherapy improves survival relatively more.

The effect of adding oxaliplatin to a fluoropyrimidine (eg, capecitabine or fluorouracil) in stage II-III colon cancer has been extensively studied.^3,27-32^ Three trials in particular have contributed to our understanding: the NO16968 (XELOX in Adjuvant Colon Cancer Treatment) study, the Multicenter International Study of Oxaliplatin/5-Fluorouracil/Leucovorin in the Adjuvant Treatment of Colon Cancer (MOSAIC) trial, and the C-07 trial of the National Surgical Adjuvant Breast and Bowel Project (NSABP).

NO16968 and MOSAIC found a small (4-6 percentage point) but significant benefit of adding oxaliplatin to the treatment regimen. In MOSAIC, treatment effect was stronger in patients with stage III compared with stage II; NO16968 only targeted stage III patients.^29,33^ Both trials included a relatively young population. NSABP, which also investigated a younger population compared with our analysis, did not find an overall benefit of adding oxaliplatin but did report a significant effect in an unplanned subset analysis of patients 70 years and younger.^32^ In a subgroup analysis, the MOSAIC study drew the complimentary conclusion that there is no additional benefit of oxaliplatin in elderly patients. This is in line with the results obtained here.

In conclusion, we have shown that structure learning elucidates underlying relationships in data, helping select which variables should be corrected for. Varying included variables and algorithms yielded (slightly) different, complementary results and identified different sets of potential confounders. HRs were similar regardless of the chosen set.

We found a strong association between adjuvant treatment with capecitabine and survival in stage III colon cancer in our cohort of patients 70 years and older. No additional benefit of adding oxaliplatin was found. As such, addition of oxaliplatin may be considered in younger patients with more advanced stage but should be avoided in elderly patients.

## Data Availability

Source code accompanying this manuscript can be found online at https://github.com/mellesies/Using-BNs-for-estimating-treatment-effect-in-CRC.

## References

[1] NKR Cijfers: Incidentie—Grafiek, 2023. https://nkr-cijfers.iknl.nl

[2] van Erning F, Janssen-Heijnen MLG, Creemers GJ, et al: Recurrence-free and overall survival among elderly stage III colon cancer patients treated with CAPOX or capecitabine monotherapy. Int J Cancer 140:224-233, 2017 27615021 10.1002/ijc.30423

[3] Tournigand C, André T, Bonnetain F, et al: Adjuvant therapy with fluorouracil and oxaliplatin in stage II and elderly patients (between ages 70 and 75 years) with colon cancer: Subgroup analyses of the Multicenter International Study of Oxaliplatin, Fluorouracil, and Leucovorin in the Adjuvant Treatment of Colon Cancer trial. J Clin Oncol 30:3353-3360, 2012 22915656 10.1200/JCO.2012.42.5645

[4] Rubin DB, Thomas N: Matching using estimated propensity scores: Relating theory to practice. Biometrics 52:249-264, 1996 8934595

[5] Rubin DB: Estimating causal effects from large data sets using propensity scores. Ann Intern Med 127:757-763, 1997 9382394 10.7326/0003-4819-127-8_part_2-199710151-00064

[6] Li J, Handorf E, Bekelman J, et al: Propensity score and doubly robust methods for estimating the effect of treatment on censored cost. Stat Med 35:1985-1999, 2016 26678242 10.1002/sim.6842 PMC4848146

[7] Genbäck M, de Luna X: Causal inference accounting for unobserved confounding after outcome regression and doubly robust estimation. Biometrics 75:506-515, 2019 30430543 10.1111/biom.13001

[8] Mantel N, Haenszel W: Statistical aspects of the analysis of data from retrospective studies of disease. J Natl Cancer Inst 22:719-748, 1959 13655060

[9] Mantel N: Chi-square tests with one degree of freedom; extensions of the Mantel-Haenszel procedure. J Am Stat Assoc 58:690-700, 1963

[10] Häggström J: Data-driven confounder selection via Markov and Bayesian Networks. Biometrics 74:389-398, 2018 29096036 10.1111/biom.12788

[11] Pearl J: Causality: Models, Reasoning and Inference (ed 2). New York, NY, Cambridge University Press, 2009

[12] VanderWeele TJ, Shpitser I: A new criterion for confounder selection. Biometrics 67:1406-1413, 2011 21627630 10.1111/j.1541-0420.2011.01619.x PMC3166439

[13] Sieswerda M, Xie S, van Rossum R, et al: Identifying confounders using Bayesian Networks and estimating treatment effect in prostate cancer with observational data. JCO Clin Cancer Inform 7:e2200080, 2023 36595730 10.1200/CCI.22.00080

[14] Koller D, Friedman N: Probabilistic Graphical Models: Principles and Techniques. Cambridge, Massachusets; London, England, MIT Press, 2009

[15] Steck H: Constraint-based structural learning in Bayesian Networks using finite data sets [dissertation]. Munich Technical University, Munich, Germany, 2001

[16] Silander T, Myllymaki P: A simple approach for finding the globally optimal Bayesian Network structure, 2012. http://arxiv.org/abs/1206.6875

[17] Tsamardinos I, Brown LE, Aliferis CF: The max-min hill-climbing Bayesian Network structure learning algorithm. Mach Learn 65:31-78, 2006

[18] Andersen SK, Olesen KG, Jensen FV, et al: HUGIN—A shell for building Bayesian Belief universes for expert systems. Proc Elev Int Jt Conf Artif Intell 2:1080-1085, 1989

[19] R Core Team: R: A language and environment for statistical computing. Vienna, Austria, 2022. https://www.R-project.org/

[20] Franzin A, Sambo F, di Camillo B: bnstruct: an R package for Bayesian Network structure learning in the presence of missing data. Bioinformatics 33:1250-1252, 2017 28003263 10.1093/bioinformatics/btw807

[21] Epskamp S, Cramer AOJ, Waldorp LJ, et al: qgraph: Network visualizations of relationships in psychometric data. J Stat Softw 48:1–18, 2012

[22] Agresti A: Categorical Data Analysis (ed 2). New York, NY, Wiley, 2002

[23] DeCosse JJ, Ngoi SS, Jacobson JS, et al: Gender and colorectal cancer. Eur J Cancer Prev 2:105-116, 1993 8461861 10.1097/00008469-199303000-00003

[24] Marija C, Kresimir D, Ognjen B, et al: Estimation of colon cancer grade and metastatic lymph node involvement using DWI/ADC sequences. Acta Radiol 64:1341-1346, 2022 36197524 10.1177/02841851221130008

[25] Quach LH, Jayamaha S, Whitehouse SL, et al: Comparison of the Charlson Comorbidity Index with the ASA score for predicting 12-month mortality in acute hip fracture. Injury 51:1004-1010, 2020 32151423 10.1016/j.injury.2020.02.074

[26] Wilkinson NW, Yothers G, Lopa S, et al: Long-term survival results of surgery alone versus surgery plus 5-fluorouracil and leucovorin for stage II and stage III colon cancer: Pooled analysis of NSABP C-01 through C-05. A baseline from which to compare modern adjuvant trials. Ann Surg Oncol 17:959-966, 2010 20082144 10.1245/s10434-009-0881-y PMC2935319

[27] Haller DG, Tabernero J, Maroun J, et al: Capecitabine plus oxaliplatin compared with fluorouracil and folinic acid as adjuvant therapy for stage III colon cancer. J Clin Oncol 29:1465-1471, 2011 21383294 10.1200/JCO.2010.33.6297

[28] André T, Boni C, Mounedji-Boudiaf L, et al: Oxaliplatin, fluorouracil, and leucovorin as adjuvant treatment for colon cancer. N Engl J Med 350:2343-2351, 2004 15175436 10.1056/NEJMoa032709

[29] André T, de Gramont A, Vernerey D, et al: Adjuvant fluorouracil, leucovorin, and oxaliplatin in stage II to III colon cancer: Updated 10-year survival and outcomes according to BRAF mutation and mismatch repair status of the MOSAIC study. J Clin Oncol 33:4176-4187, 2015 26527776 10.1200/JCO.2015.63.4238

[30] Aparicio T, Francois E, Cristol-Dalstein L, et al: PRODIGE 34-FFCD 1402-ADAGE: Adjuvant chemotherapy in elderly patients with resected stage III colon cancer: A randomized phase 3 trial. Dig Liver Dis 48:206-207, 2016 26748426 10.1016/j.dld.2015.11.023

[31] Aparicio T, Bouché O, Etienne P-L, et al: Preliminary tolerance analysis of adjuvant chemotherapy in older patients after resection of stage III colon cancer from the PRODIGE 34-FFCD randomized trial. Dig Liver Dis 55:541-548, 2022 36115817 10.1016/j.dld.2022.08.036

[32] Yothers G, O’Connell MJ, Allegra CJ, et al: Oxaliplatin as adjuvant therapy for colon cancer: Updated results of NSABP C-07 trial, including survival and subset analyses. J Clin Oncol 29:3768-3774, 2011 21859995 10.1200/JCO.2011.36.4539 PMC3188282

[33] Schmoll H-J, Tabernero J, Maroun J, et al: Capecitabine plus oxaliplatin compared with fluorouracil/folinic acid as adjuvant therapy for stage III colon cancer: Final results of the NO16968 randomized controlled phase III trial. J Clin Oncol 33:3733-3740, 2015 26324362 10.1200/JCO.2015.60.9107

